# Transcarotid transcatheter aortic valve replacement combined with percutaneous coronary intervention for severe aortic stenosis with coronary artery disease in a tortuous aortic arch: a Case Report

**DOI:** 10.3389/fcvm.2025.1522100

**Published:** 2025-05-16

**Authors:** Wenwen Chen, Yue Bao, Hui Guo

**Affiliations:** Department of Cardiology, Wuhan Asia Heart Hospital, Wuhan, China

**Keywords:** transcatheter aortic valve replacement, percutaneous coronary intervention, transcarotid, aortic tortuosity, aortic stenosis

## Abstract

Transcatheter Aortic Valve Replacement (TAVR) is currently the preferred treatment option not only for high-surgical-risk patients with severe aortic stenosis (AS) but also increasingly for those with intermediate and low surgical risk. Coronary artery disease (CAD) is one of the most common complications in severe AS patients, making percutaneous coronary intervention (PCI) a frequent clinical requirement in cases of severe AS complicated by CAD. The coexistence of Aortic Tortuosity and aortic stenosis is extremely rare.We report a case of an elderly male with severe aortic stenosis combined with coronary artery disease, who underwent successful transcarotid TAVR and PCI due to a Z-shaped fold tortuosity in the aortic arch, making femoral access challenging. The patient was able to ambulate the following day and was discharged on the fifth postoperative day with stable follow-up. As of this writing, no related reports of combined transcarotid TAVR and PCI have been published.

## Introduction

Aortic stenosis (AS) is a common valvular heart disease with a poor prognosis. With increasing evidence supporting the efficacy of TAVR in intermediate- and low-risk AS patients, TAVR is becoming a viable treatment option for more patients with aortic valve disease (1, 2). Coronary artery disease (CAD) is a common comorbidity in patients undergoing TAVR, with a prevalence of about 50% that increases with age and impacts treatment strategy and prognosis (3–5). While one-stage TAVR combined with PCI has shown clinical success, the superiority of simultaneous vs. staged intervention remains controversial and requires further data (3, 6–9). Although TAVR typically uses a transfemoral approach, alternative access routes can be chosen if femoral access is challenging. Herein, we present a case of severe AS with CAD in an elderly male, treated with transcarotid TAVR combined with PCI due to a tortuous aortic arch, the first reported case of this kind as of this writing, no related reports have been published.

## Case report

A 77-year-old male, height 159 cm, weight 58 kg, was admitted to Wuhan Asia Heart Hospital on June 6, 2024, with a 2-year history of intermittent chest tightness and palpitations, worsened over the prior two weeks. Previous treatments at another hospital for coronary artery disease included rotational atherectomy and drug-coated balloon angioplasty of the left anterior descending artery. The patient had a 10-year history of diabetes mellitus. Auscultation of the first aortic valve area revealed a grade 3/6 systolic murmur.

N-terminal pro-B-type natriuretic peptide (NT-proBNP) was 7,417.00 pg/ml and Hyper-sensitive cardiac troponin I (hs-cTnI)was 0.0766 ng/ml. Electrocardiography (ECG) showed atrial flutter with a rapid ventricular response, and ST-T segment changes. Transthoracic echocardiography (TTE) showed: Type 0 bicuspid aortic valve with severe stenosis and mild-to-moderate regurgitation, regional wall motion abnormalities in the left ventricle, mild-to-moderate mitral regurgitation, ascending aortic dilation, interventricular septal thickening, reduced left ventricular systolic function, left ventricular end-diastolic diameter of 5.7 cm, left ventricular outflow tract diameter of 2.5 cm, ejection fraction of 45%, peak aortic valve velocity of 4.9 m/s, peak transvalvular pressure gradient of 95 mmHg, mean transvalvular pressure gradient of 57 mmHg, and aortic valve area of 0.6 cm². Chest computed tomography (CT) showed dilation of the ascending aorta, and atherosclerosis in the thoracic aorta, with a local Z-shaped tortuosity in the descending aorta. He was diagnosed with severe AS complicated with CAD.

Preoperative CT indicated that the aortic valve has a Type 0 L-R bicuspid configuration with a horizontal alignment. The annulus has an average diameter of 25.2 mm, and the outflow tract measures 24 mm. The calcification score is 1,063 mm³, with calcification mainly located on the leaflets, commissures, and root. The leaflets are thickened, with calcification in the left anterior descending (LAD) artery. Coronary ostium height is adequate, presenting a low risk of obstruction, and the heart shows a horizontal orientation at a 71° angle.

**Figure 1:**
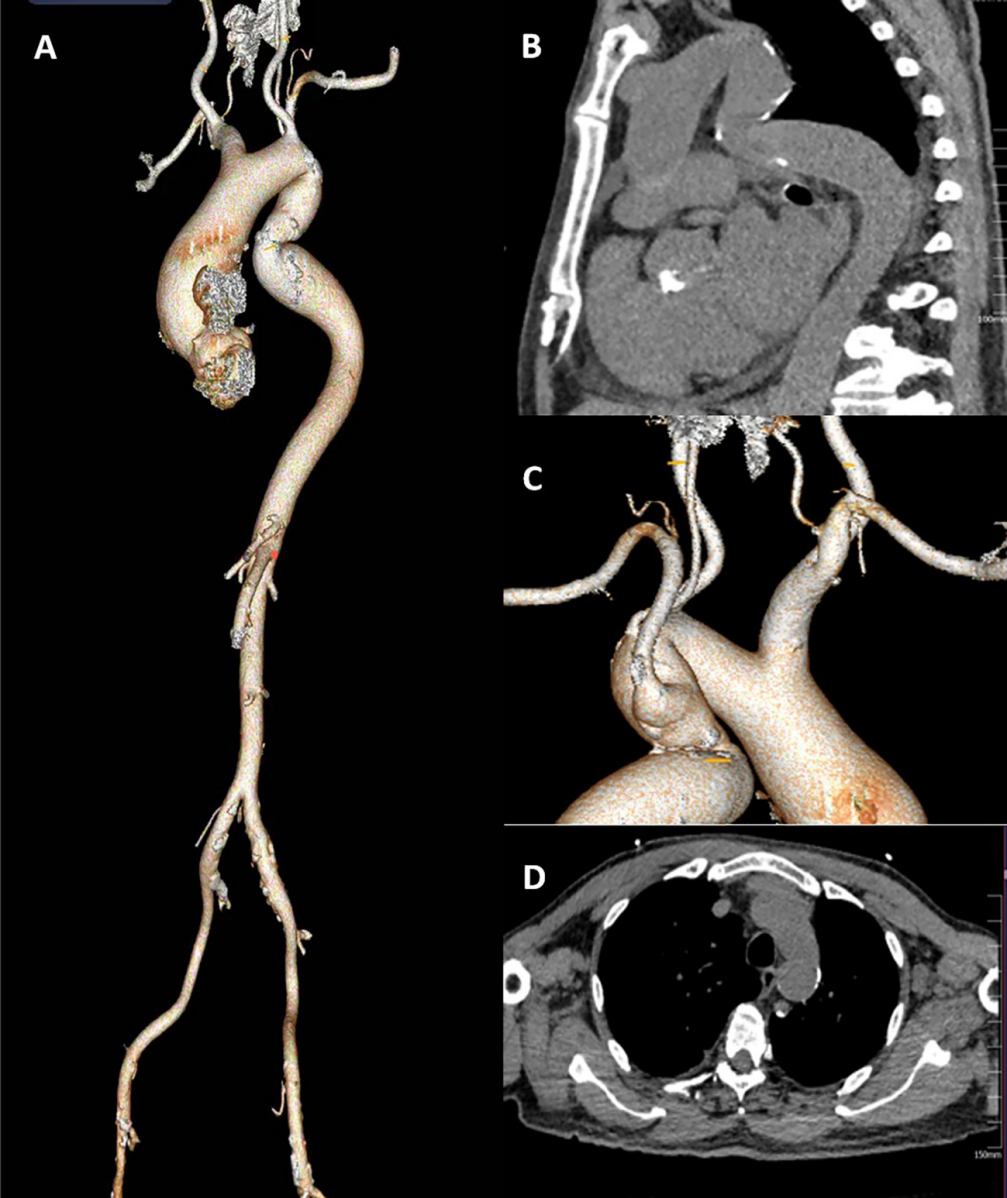
**(A)** the full CT of the aorta. **(B)** a Z-shaped fold tortuosity in the descending aortic arch.**(C)** the left common carotid artery is free from stenosis or calcification, the Willis circle is complete. The right vertebral artery is dominant. **(D)** The narrowest segment of the descending aorta).

This patient had severe AS combined with CAD*.* His descending aortic arch has a localized tortuosity (Figures 1A,B), while the left common carotid artery is free from stenosis or calcification, and the Willis circle is complete. The right vertebral artery is dominant, and the pathway from the left common carotid artery to the annulus is feasible, the left subclavian artery was relatively small in diameter, and the right subclavian artery showed marked angulation (Figure 1C). Chest CT demonstrated a minimal descending aortic diameter of 21 mm at the narrowest segment, with no significant caliber change compared to adjacent normal segments and no evidence of collateral vessel formation (Figure 1D). Following Multi-Disciplinary Team (MDT) discussion and with the consent of the family, a transcarotid, one-stage TAVR procedure combined with PCI was planned.

**Figure 2:**
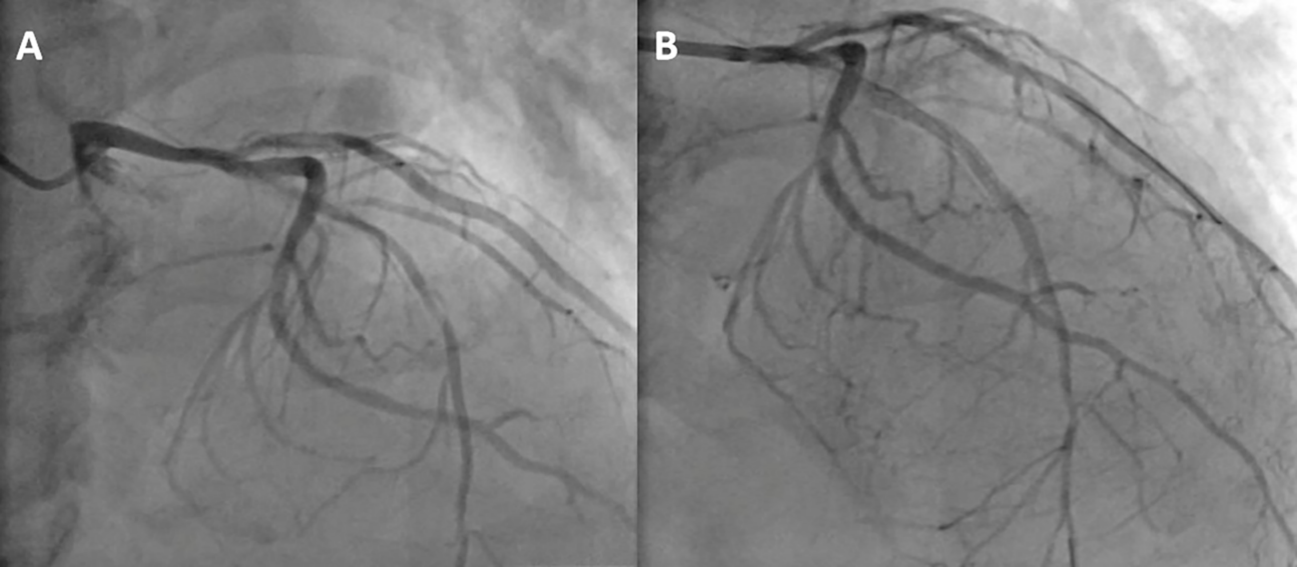
**(A)** CAG indicating a 90% diffuse stenosis in the proximal segment of the LAD artery. **(B)** the LAD artery after stent implantation).

On June 8, 2024, right radial artery access was obtained under local anesthesia with 1% lidocaine. Coronary angiography (CAG) revealed 90% diffuse stenosis in the proximal left anterior descending (LAD) artery and 70% segmental stenosis in the Diagonal Branches(D) (Figure 2A). Intravascular ultrasound (IVUS) revealed severe calcified lesions in the proximal LAD. A shockwave balloon (3.0  ×  12 mm) was deployed in the proximal LAD, delivering 80 pulses, followed by the implantation of three drug-eluting stents (2.5 mm  ×  33 mm, 2.75 mm  ×  26 mm, and 3.0 mm  ×  15 mm). IVUS assessment confirmed good stent apposition, with a minimal stent area (MSA) greater than 6.0 mm² (Figure 2B).

**Figure 3:**
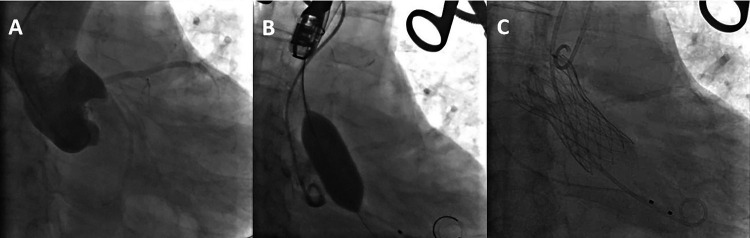
**(A)** the aortic root angiography. **(B)** balloon expansion **(C)** the completion of artificial valve deployment.

After PCI, general anesthesia was administered for the TAVR procedure. A temporary pacemaker was inserted via the right internal jugular vein. The left internal carotid artery was selected as the primary access route, with the right radial artery as a secondary route. Through the right radial artery sheath, a pigtail catheter was advanced into the ascending aorta for aortography (Figure 3A). An AL2 catheter was introduced through the sheath of the left internal carotid artery into the ascending aorta. A straight guidewire was navigated across the aortic valve into the left ventricle, and the AL2 catheter was exchanged for a pigtail catheter to measure the left ventricular and aortic pressures, yielding a left ventricular systolic/diastolic pressure of 216/17 mmHg and an aortic pressure of 105/61 mmHg. With the stiffened guidewire positioned in the left ventricle, the pigtail catheter was exchanged for a delivery catheter, oriented appropriately for the procedure. A 23 mm  ×  4 cm NUMED balloon was used for pre-dilation of the aortic valve under rapid pacing at 180 bpm to stabilize the valve area during dilation(Figure 3B). Aortography was conducted to assess the risk of coronary obstruction and valve regurgitation. Based on preoperative CT scans, echocardiography measurements, and the results of the balloon dilation, a 29 mm Peijia TAVR prosthesis was selected and loaded onto the delivery system, then advanced over the stiffened guidewire into the left ventricle. Under guidance from digital subtraction angiography (DSA) and Transesophageal echocardiography (TEE), and while maintaining rapid pacing at 180 bpm, the prosthetic valve was gradually deployed (Figure 3C). After release, the delivery system was withdrawn. Post-procedure measurements indicated a peak gradient across the valve of 9 mmHg. Aortography of the aortic root showed no paravalvular leak or coronary obstruction. The depth of the implanted valve was measured at 3 mm on the non-coronary cusp and 6 mm on the left coronary cusp. TEE revealed mild central regurgitation with no paravalvular leak. Follow-up measurements demonstrated left ventricular and aortic pressures of 120/20 mmHg and 120/70 mmHg, respectively. Continuous cardiac monitoring showed sinus rhythm with no conduction block, QRS widening, or PR prolongation. The delivery system and arterial sheath were removed, and surgical closure of the left internal carotid artery was performed, along with the removal of the right radial artery sheath while maintaining the temporary pacemaker.

**Figure 4:**
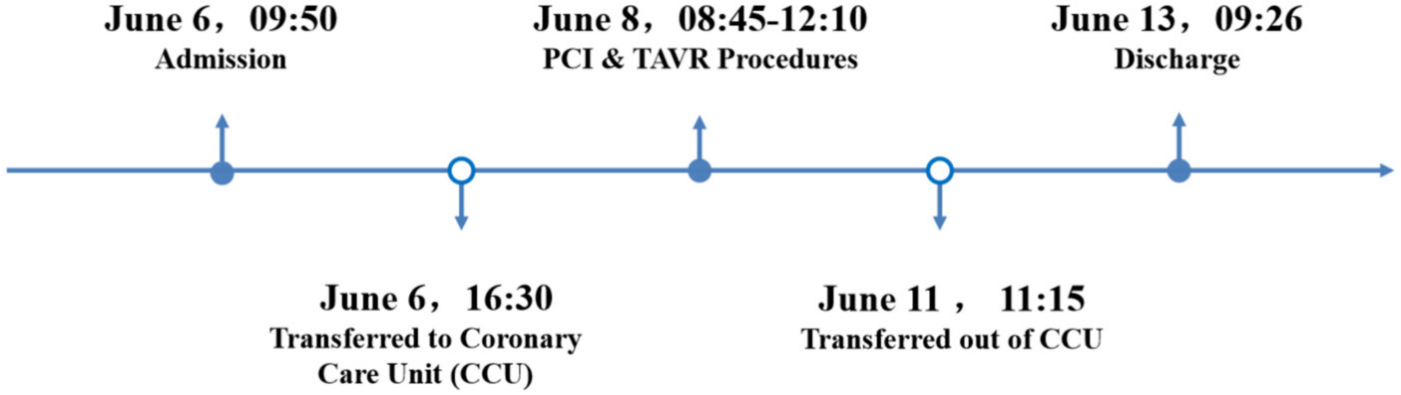
A timeline with relevant data from the episode of care.

The patient's vital signs remained stable after surgery. Holter monitoring showed sinus rhythm with occasional episodes of atrial flutter and atrial fibrillation. TTE indicated normal function of the implanted valve and mildly reduced left ventricular systolic function. The patient was managed postoperatively with anticoagulants, antiplatelet agents, diuretics to improve cardiac function, lipid-lowering therapy, ventricular remodeling inhibitors, glucose-lowering agents, and renal protection. The patient was able to ambulate the following day and was discharged five days postoperatively. A one-month follow-up revealed no symptoms of chest tightness or palpitations. ECG showed sinus rhythm with frequent premature atrial contractions, and TTE confirmed normal function of the implanted valve with mildly reduced left ventricular systolic function. At three months and six months post-op, all follow-up evaluations were normal. The patient's hospitalization timeline is shown in Figure 4.

## Discussion

This case involved a patient with severe aortic stenosis complicated by chronic coronary artery disease who underwent Transcarotid TAVR combined with PCI due to a Z-shaped fold tortuosity of the aorta.

The patient exhibited pronounced tortuosity and angulation of the descending aorta. Although successful TAVR using femoral or axillary artery access has been reported for patients with tortuous aortas, these approaches require specialized equipment and techniques (10, 11). Along with significant curvature of the aortic arch. Based on our previous experience, transfemoral access would carry a high risk of intraoperative acute aortic injury, even with the use of longer sheaths or the placement of an additional stiff guidewire via an auxiliary access to enhance support. Although bilateral subclavian access could potentially minimize the impact on cerebral perfusion, anatomical limitations were present: the left subclavian artery was relatively small in diameter, and the right subclavian artery showed marked angulation. Following multidisciplinary team (MDT) consultation and evaluation of procedural feasibility, left carotid artery access was selected Transcarotid TAVR, as an alternative route to the transfemoral approach, has been confirmed in numerous clinical studies to safely and effectively perform TAVR with favorable outcomes, including lower rates of stroke and major vascular complications (12, 13). Preoperative CT angiography for this patient included evaluation of the aortic root, coronary ostial height, carotid artery access, and cerebrovascular assessment (including the integrity of the Willis circle). Intraoperatively, the left carotid artery was surgically exposed, and a 10F arterial sheath was initially inserted. Through this sheath, a guidewire was advanced across the native aortic valve and subsequently exchanged for a stiff guidewire positioned in the left ventricle. A 20F large-bore sheath was then introduced to facilitate subsequent balloon aortic valvuloplasty (BAV) and TAVR, with the goal of minimizing interruption of cerebral blood flow. The duration of left carotid artery occlusion by the 20F sheath was limited to 18 min. Throughout the procedure, continuous cerebral oximetry monitoring was performed by the anesthesia team to closely assess and ensure adequate cerebral perfusion, and there were no postoperative complications such as vascular bleeding, dissection, stroke, or atrioventricular conduction block.

Several studies have shown the safety and efficacy of one-stage treatment using TAVR combined with PCI. It is necessary, however, to monitor the risks of renal damage and bleeding carefully and to comprehensively consider the patient's overall condition, disease severity, possible complications, and surgical risk to develop an individualized treatment plan (3, 7, 8, 14). This patient presented with severe aortic stenosis and coronary artery disease. The PCI procedure was performed on the proximal left anterior descending (LAD) artery via the right radial artery, with intravascular ultrasound (IVUS) revealing severe calcification, which was treated with a shockwave balloon before implanting three drug-eluting stents. TAVR was then performed using the left internal carotid artery as the primary access and the right radial artery as an auxiliary route, with the Peijia TAV29 transcatheter valve being deployed. The patient was mobilized the next day and discharged on postoperative day 5, with follow-up examinations showing stable results, indicating successful surgery.

As of this writing, no related reports of combined transcarotid TAVR and PCI have been published. With the growing body of evidence supporting TAVR, improved surgical accessibility, and advances in valve technology, transcarotid TAVR combined with PCI may become a viable option for more patients with aortic valve disease complicated by chronic coronary artery disease who have difficulty with femoral access. A thorough preoperative examination, comprehensive assessment of the patient's condition, individualized treatment planning, and meticulous surgical strategy can further ensure favorable clinical outcomes for this procedure. However,the current single-patient experience at our institution limits definitive conclusions regarding this method's safety and efficacy. Additional studies are needed to establish indications, standardize the procedure, and enhance its effectiveness through iterative refinements.

## Data Availability

The original contributions presented in the study are included in the article/Supplementary Material, further inquiries can be directed to the corresponding author.

## References

[1] Mack MJ Leon MB Thourani VH Makkar R Kodali SK Russo M Transcatheter aortic-valve replacement with a balloon-expandable valve in low-risk patients. N Engl J Med. (2019) 380(18):1695–705. 10.1056/NEJMoa1814052 30883058

[2] Popma JJ Deeb GM Yakubov SJ Mumtaz M Gada H O’Hair D Transcatheter aortic-valve replacement with a self-expanding valve in low-risk patients. N Engl J Med. (2019) 380(18):1706–15. 10.1056/NEJMoa1816885 30883053

[3] Faroux L Guimaraes L Wintzer-Wehekind J Junquera L Ferreira-Neto AN del Val D Coronary artery disease and transcatheter aortic valve replacement: JACC state-of-the-art review. J Am Coll Cardiol. (2019) 74(3):362–72. 10.1016/j.jacc.2019.06.012 31319919

[4] Otto CM Nishimura RA Bonow RO Carabello BA Erwin JP Gentile F 2020 ACC/AHA guideline for the management of patients with valvular heart disease: executive summary: a report of the American College of Cardiology/American Heart Association joint committee on clinical practice guidelines. Circulation. (2021) 143(5):e35–71. 10.1161/CIR.0000000000000932 33332149

[5] Tarantini G Tang G Nai Fovino L Blackman D Van Mieghem NM Kim W-K Management of coronary artery disease in patients undergoing transcatheter aortic valve implantation. A clinical consensus statement from the European association of percutaneous cardiovascular interventions in collaboration with the ESC working group on cardiovascular surgery. EuroIntervention. (2023) 19(1):37–52. 10.4244/EIJ-D-22-00958 36811935 PMC10174192

[6] Xu C Hu H Su X. Concomitant percutaneous coronary intervention and transcatheter aortic valve replacement for aortic stenosis complicated with acute STEMI: a case report and literature review. Front Cardiovasc Med. (2023) 10:1291089. 10.3389/fcvm.2023.1291089 38089776 PMC10713811

[7] Fischer J Steffen J Arlart T Haum M Gschwendtner S Doldi PM Concomitant percutaneous coronary intervention in patients undergoing transcatheter aortic valve implantation. Catheter Cardiovasc Interv. (2024) 103(1):186–93. 10.1002/ccd.30927 38140761

[8] Park DY Simonato M Ahmad Y Banks AZ Lowenstern A Nanna MG. Insight into the optimal timing of percutaneous coronary intervention and transcatheter aortic valve replacement. Curr Probl Cardiol. (2024) 49(1 Pt A):102050. 10.1016/j.cpcardiol.2023.102050 37643698 PMC10924682

[9] Ochiai T Yoon S-H Flint N Sharma R Chakravarty T Kaewkes D Timing and outcomes of percutaneous coronary intervention in patients who underwent transcatheter aortic valve implantation. Am J Cardiol. (2020) 125(9):1361–8. 10.1016/j.amjcard.2020.01.043 32106928

[10] Yang Y Yang H Pan J Zhang G. Strategies to address extreme aortic tortuosity during transcatheter aortic valve replacement. JACC Cardiovasc Interv. (2022) 15(7):791–2. 10.1016/j.jcin.2022.01.305 35305909

[11] Harloff MT Percy ED Hirji SA Yazdchi F Shim H Chowdhury M A step-by-step guide to trans-axillary transcatheter aortic valve replacement. Ann Cardiothorac Surg. (2020) 9(6):510–21. 10.21037/acs-2020-av-79 33312914 PMC7724056

[12] Bob-Manuel T Almusawi H Rezan T Khaira H Akingbola A Nasir A Efficacy and safety of transcarotid transcatheter aortic valve replacement: a systematic review. Cardiovasc Revasc Med. (2020) 21(7):917–26. 10.1016/j.carrev.2019.12.012 31882332

[13] Chamandi C Abi-Akar R Rodés-Cabau J Blanchard D Dumont E Spaulding C Transcarotid compared with other alternative access routes for transcatheter aortic valve replacement. Circ Cardiovasc Interv. (2018) 11(11):e006388. 10.1161/CIRCINTERVENTIONS.118.006388 30571205

[14] Ling Ma Jing-ya Wang Qian-wei Xu Xiao-lin Yu. Research progress on treatment strategies for simultaneous/staged percutaneous coronary intervention in patients with severe aortic valve stenosis and chronic coronary artery disease undergoing percutaneous aortic valve replacement surgery. Chin J Intervent Cardiol. (2024) 32(3):271–5. 10.3969/j.issn.1004-8812.2024.05.007

