# Supplementary figures and images for: Transcarotid transcatheter aortic valve replacement combined with percutaneous coronary intervention for severe aortic stenosis with coronary artery disease in a tortuous aortic arch: a Case Report
Source: Front Cardiovasc Med. 2025 May 16;12:1522100. doi: 10.3389/fcvm.2025.1522100 (PMC12122426; doi:10.3389/fcvm.2025.1522100)

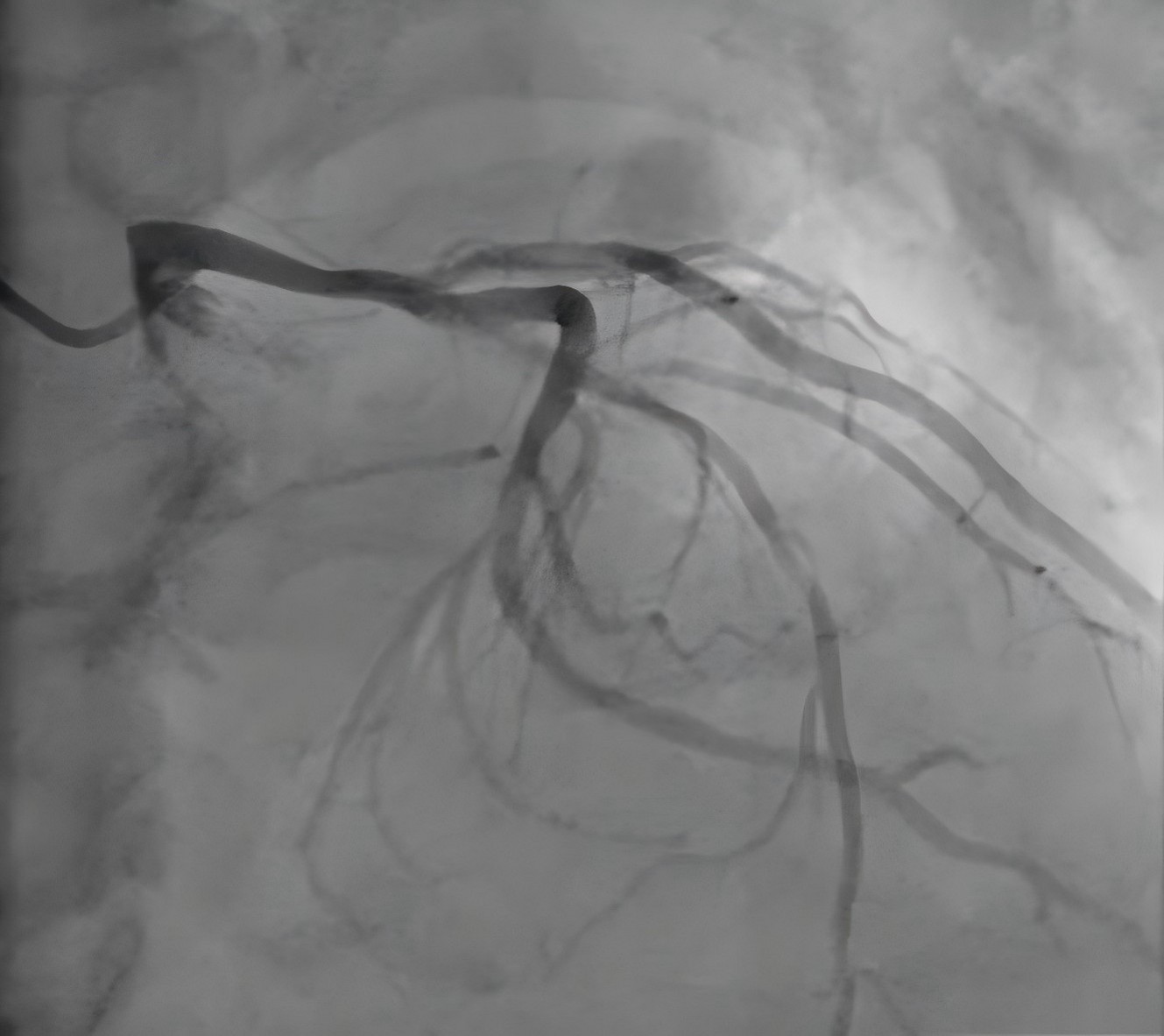

Supplement: Supplementary file 1 [file Image1.jpeg]

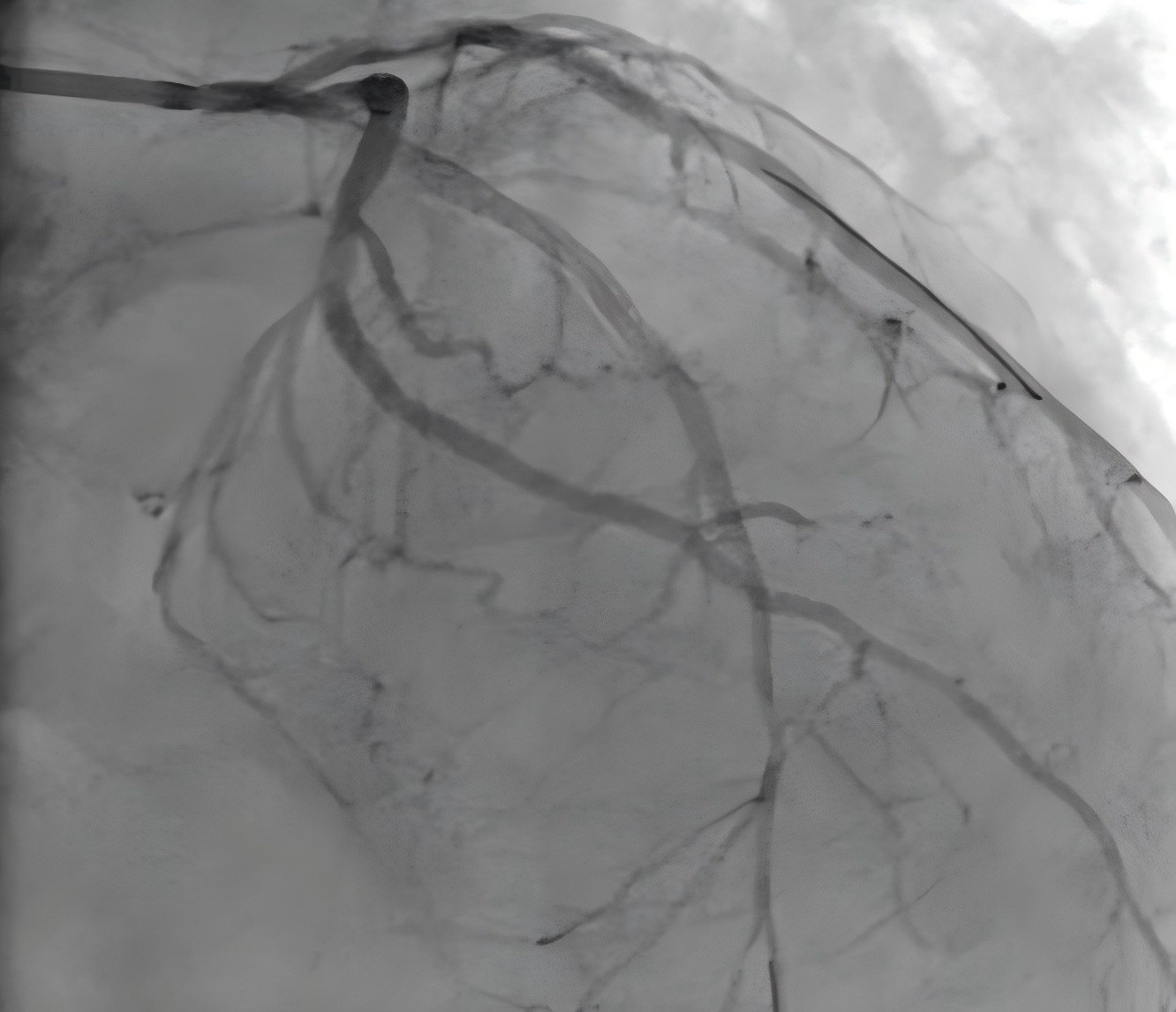

Supplement: Supplementary file 2 [file Image2.jpeg]

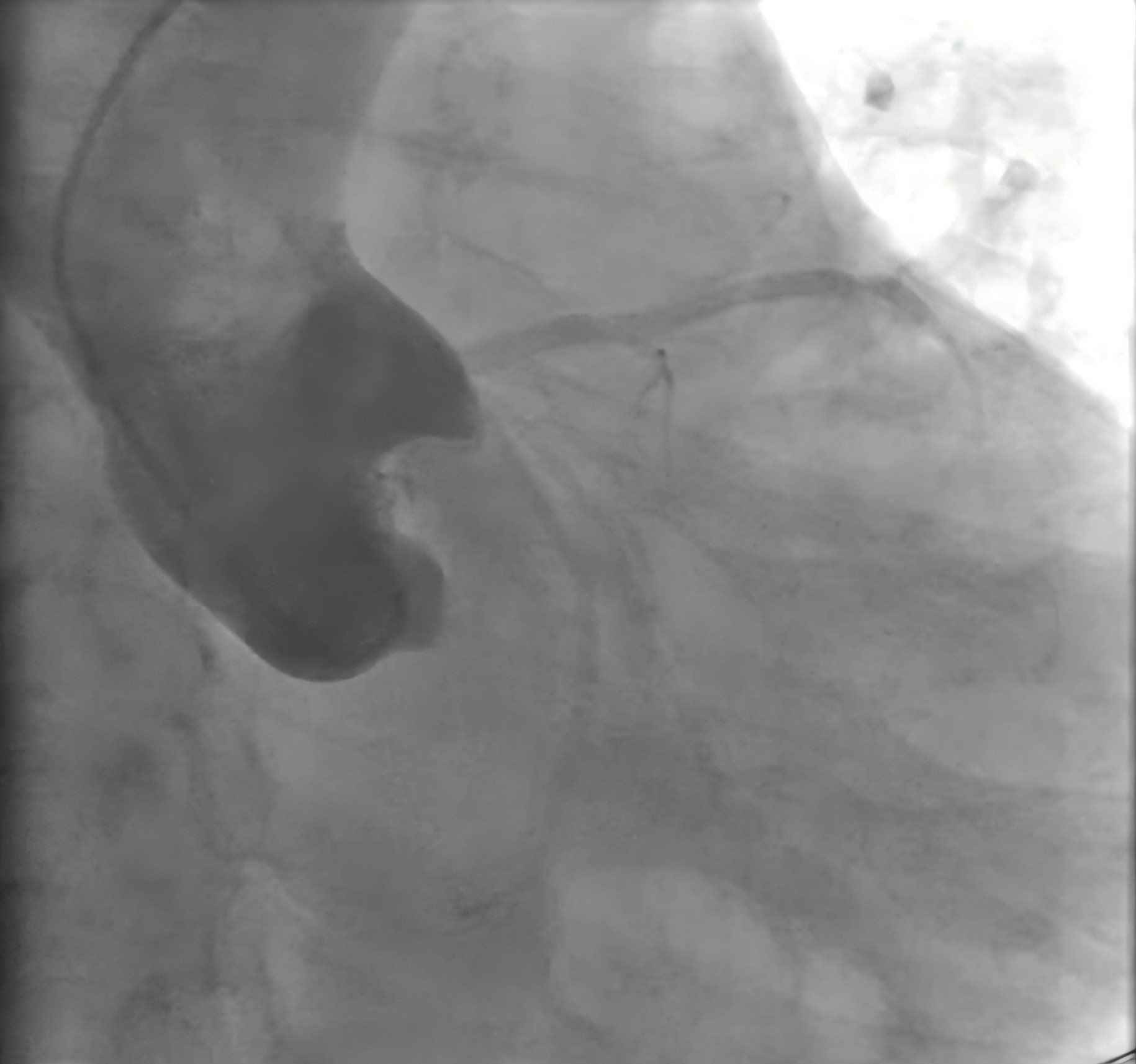

Supplement: Supplementary file 3 [file Image3.jpeg]

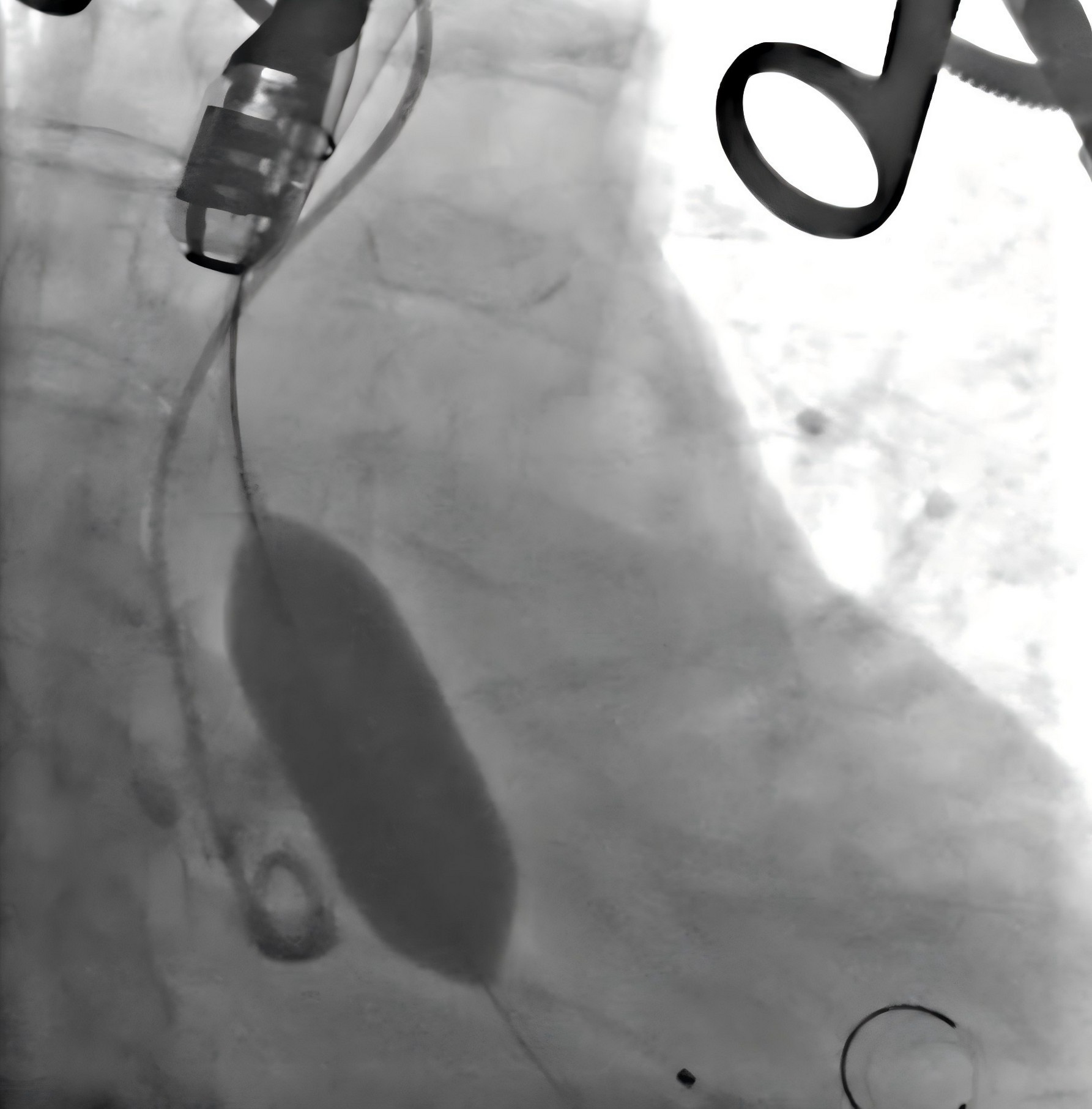

Supplement: Supplementary file 4 [file Image4.jpeg]

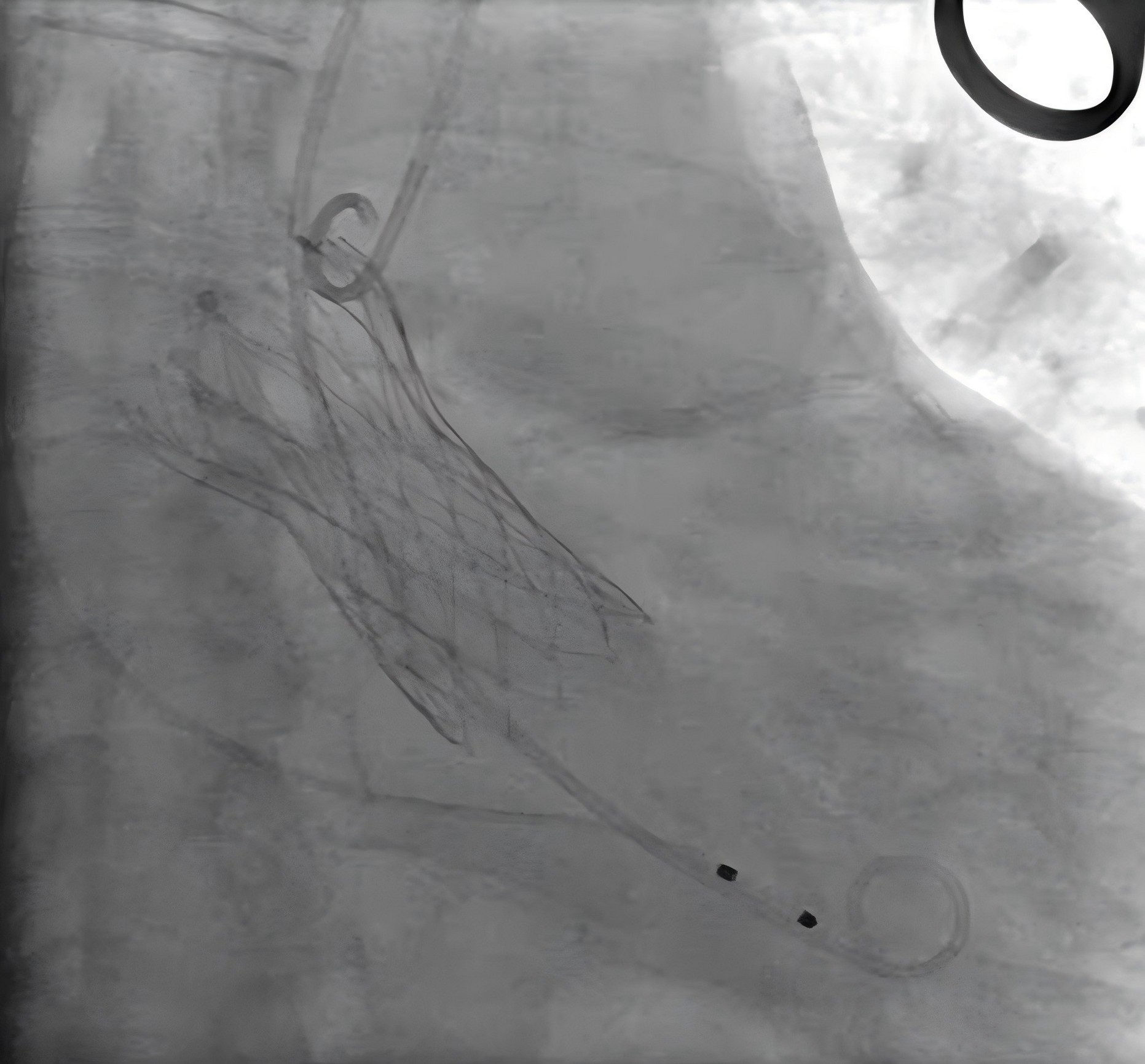

Supplement: Supplementary file 5 [file Image5.jpeg]

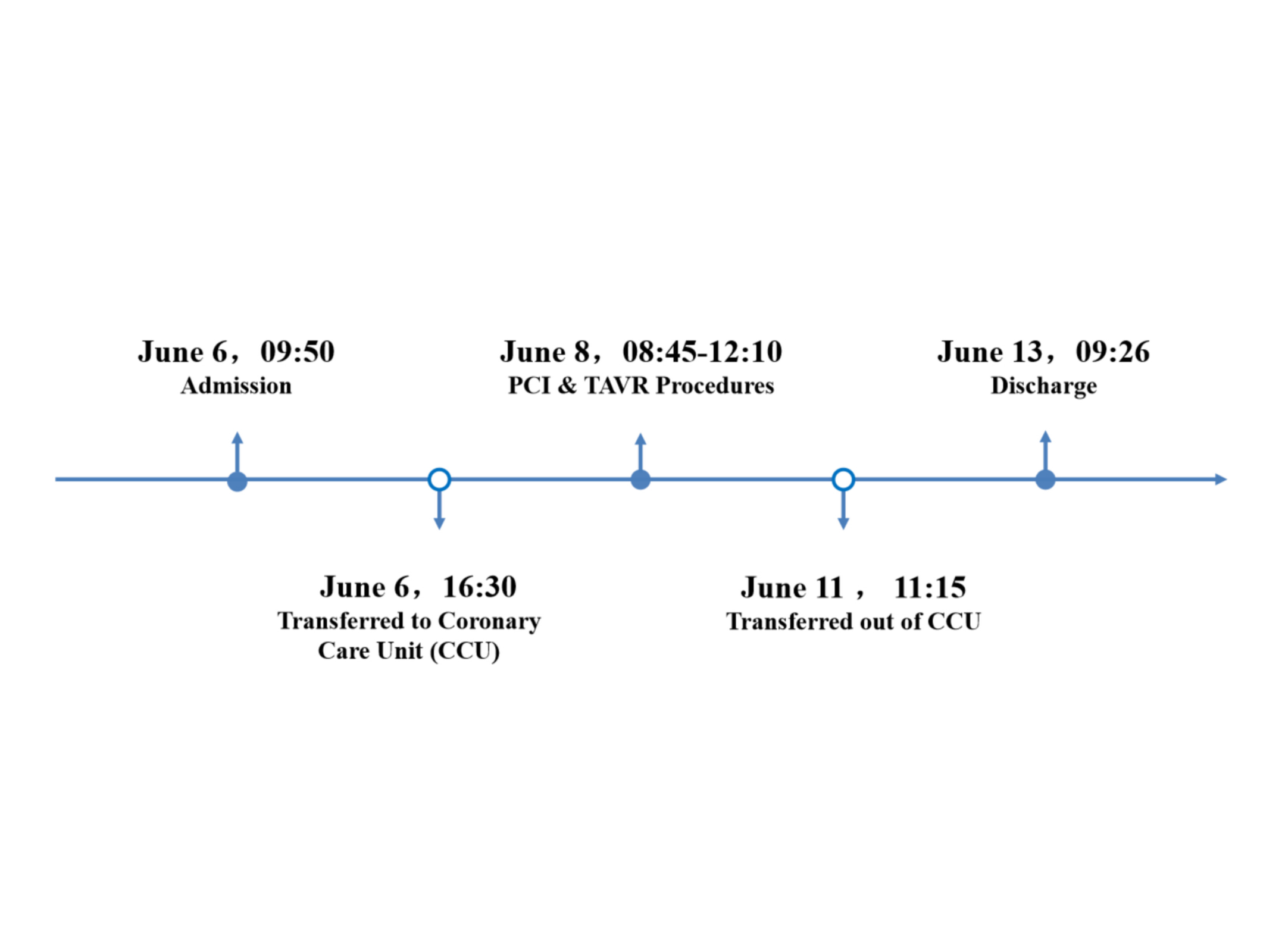

Supplement: Supplementary file 6 [file Image6.jpeg]
